# Dyslipidemia Associated With Type 2 Diabetes Mellitus as a Strong Predictor of ICU Admission in COVID-19 Patients: A Retrospective Comparative Study

**DOI:** 10.7759/cureus.87193

**Published:** 2025-07-02

**Authors:** Abdurahman Alfaiz, Mohammad Shabbir, Mohammad D Alotaibi, Feras Almarshad, Muath A Alammar, Zeeshan Ahsan

**Affiliations:** 1 Clinical Sciences, College of Medicine, Shaqra University, Shaqra, SAU; 2 Internal Medicine, College of Medicine, Shaqra University, Shaqra, SAU; 3 Family Medicine, College of Medicine, Shaqra University, Shaqra, SAU; 4 Dentistry, Epidemiology and Biostatistics, Dow University of Health Sciences, Karachi, PAK

**Keywords:** covid-19, diabetes mellitus, dyslipidemia, icu, predictors

## Abstract

Background: The global COVID-19 pandemic has raised questions about the complex interplay between chronic non-communicable diseases and the clinical trajectory of the disease. Among these, dyslipidemia and type 2 diabetes mellitus (T2DM) have emerged as significant contributors to adverse COVID-19 outcomes and intensive care unit (ICU) admissions. This retrospective comparative study was conducted at Prince Mohammed bin Abdulaziz Hospital in Riyadh, Kingdom of Saudi Arabia (KSA), and assessed the impact of dyslipidemia and T2DM on COVID-19 severity and ICU admission.

Methodology: A retrospective comparative study analyzed the data of 239 COVID-19 patients admitted to Prince Mohammed bin Abdulaziz Hospital in Riyadh between March and July 2020. The study assessed COVID-19 severity among patients with dyslipidemia and T2DM and the need for ICU admission. Data analysis employed odds ratios, Fisher's exact tests, and binary logistic regression using SPSS version 25 (IBM, SPSS, Chicago, IL, USA).

Results: The findings revealed a very strong association between dyslipidemia and T2DM coexistence (adjusted OR (aOR) = 120.82, P = 0.001). Dyslipidemia significantly increased the risk of tachypnea (aOR = 58.263, P = 0.003), shortness of breath (SoB) (aOR = 10.729 (1.251-92.016), P = 0.003), and the likelihood of ICU admission, independently, as well as in the presence of T2DM (aOR = 8.136, P = 0.026). The results showed a significant association with abnormal high-density lipoprotein (HDL) cholesterol, triglycerides, and total cholesterol (P < 0.05). Fever is notably linked to dyslipidemia (P = 0.048). Among inflammatory markers, fibrinogen showed a very strong association with dyslipidemia in the presence of T2DM, in the context of the ICU setting (1,235 (10.021-152,261.746), P = 0.004). Similarly, chest X-ray (CXR) findings also showed significant association with dyslipidemia (P = 0.039).

Conclusion: This study highlights the substantial impact of dyslipidemia, independently, as well as in the presence of T2DM, on ICU admission among COVID-19 patients in Riyadh, KSA. The findings emphasize the need for tailored care, early intervention, and optimized management of comorbid conditions. Proactive risk assessment in individuals with T2DM and dyslipidemia can reduce the probability of severe illness and ICU admission in COVID-19 patients.

## Introduction

COVID-19 pandemic, caused by the novel coronavirus severe acute respiratory syndrome coronavirus 2 (SARS-CoV-2), has fundamentally altered the course of contemporary healthcare and has raised compelling questions about the factors that determine the severity and clinical trajectory of the disease. While its primary manifestations include respiratory symptoms, an array of comorbidities have been recognized as amplifiers of disease severity, including cardiovascular diseases, diabetes, dyslipidemia, hypertension, and obesity [1,2]. The intersection of these chronic non-communicable diseases with COVID-19 pandemic has unveiled a complex web of associations that require scrutiny.

Among these conditions, dyslipidemia and type 2 diabetes mellitus (T2DM) have emerged as significant contributors to adverse COVID-19 outcomes [3-5]. T2DM stands out as a particularly pertinent risk factor, due to its high prevalence globally and its known impact on the immune system and inflammatory response [6]. Research has shown that individuals with T2DM are more likely to experience severe forms of the disease and have a higher risk of complications and mortality [3]. Moreover, T2DM often coexists with other conditions like obesity and hypertension, further complicating the clinical picture [7]. Dyslipidemia, characterized by abnormal levels of lipids (cholesterol and triglycerides) in the blood, is another prevalent chronic condition often present alongside T2DM. While closely linked with T2DM, it is an independent risk factor for cardiovascular diseases and a potential contributor to a pro-inflammatory state in the body [8,9]. Therefore, it is reasonable to suspect that the combination of T2DM and dyslipidemia may have an even greater impact on COVID-19 outcomes than either condition alone. This association is not coincidental, as T2DM and dyslipidemia share common risk factors and underlying pathophysiological mechanisms. Both conditions contribute to chronic inflammation, endothelial dysfunction, and oxidative stress, which can predispose individuals to cardiovascular diseases and other complications [4,10,11]. However, despite their close pathophysiology, they exist separately as well. As such, the study of their effects independently, as well as in combination, in the presence of COVID-19 is of relevance when examining their potential role in shaping disease outcomes.

The burden of T2DM and dyslipidemia in Riyadh, Kingdom of Saudi Arabia (KSA), cannot be underrated. The prevalence of T2DM in Saudi Arabia is among the highest globally, with estimates suggesting that nearly one-fifth adults in the country are affected [12,13]. Additionally, dyslipidemia is a common comorbidity in T2DM patients [14]. As both conditions independently predispose individuals to a range of complications, understanding their collective impact on COVID-19 prognosis is of paramount importance. Thus, it is crucial to examine the local dynamics of the pandemic, considering the prevalence of T2DM and dyslipidemia.

The primary objective of this retrospective study was to assess the association between dyslipidemia, type 2 diabetes mellitus (T2DM), and the severity of COVID-19 specifically their roles in predicting intensive care unit (ICU) admissions in Riyadh, KSA. A secondary objective was to examine the individual and combined effects of abnormal lipid profiles and T2DM on respiratory distress markers and inflammatory responses in COVID-19 patients. By comparing COVID-19 cases between patients with and without these conditions, as well as assessing the need for intensive care unit (ICU) admission, we aim to provide a comprehensive evaluation of their influence. Additionally, we will explore the implications of this research for public health policies and clinical management strategies in Riyadh and, by extension, for similar populations facing the challenges of COVID-19 in other regions. It will help improve the allocation of resources, patient management, and public health interventions in more effective and efficient way.

## Materials and methods

Methodology

Study design and setting

This retrospective comparative analysis explores the relationship between dyslipidemia and type 2 diabetes mellitus (T2DM) and their impact on the prognosis of COVID-19. The study was conducted at Prince Mohammed Bin Abdulaziz Hospital (PMAH) in Riyadh, Saudi Arabia, from March 15, 2020, to July 15, 2020.

Study population

Data was collected from the medical records of patients admitted to PMAH with confirmed COVID-19 infection. Exclusion criteria included individuals with diminished mental capacity, pregnant women, and children. Diagnosis of SARS-CoV-2 infection followed guidelines set by the Saudi Center for Disease Prevention and Control. Dyslipidemia was assessed as a key variable in this study, examining its potential impact on the prognosis of COVID-19 and its association with clinical outcomes among patients with T2DM and COVID-19 infection. Dyslipidemia patients were classified based on documented medical records and the use of statins and/or lipid profile as low-density lipoprotein (LDL) (≥4.11 mmol/L), triglyceride (≥ 2.25 mmol/L), high-density lipoprotein (HDL) (<1.03 mmol/L), and total cholesterol (≥6.2 mmol/L) upon admission. Patients without a history or documentation of dyslipidemia or statins use, with normal lipid profile LDL (<3.36 mmol/L), triglyceride (<1.69 mmol/L), HDL (>1.55 mmol/L), and total cholesterol (<5.2 mmol/L) upon admission were considered normal. T2DM was defined based on documented medical records or the use of anti-diabetic medication. A hemoglobin A1c (HbA1c) reading of ≥ 6.5 and a fasting glucose of ≥ 7.0 mmol/L upon admission were considered as diabetes. Patients without history or documentation of diabetes mellitus or anti-diabetic medication, with an HbA1c reading of < 6.5 and a fasting glucose of < 7.0 mmol/L upon admission, were considered non-diabetic.

Data collection

Clinical parameters, including shortness of breath (SoB), tachypnea, fever, cough, chest pain, T2DM, and intensive care unit (ICU) admission, were recorded. Additionally, data on laboratory test results for inflammatory markers like fibrinogen, D-dimer, and C-reactive protein (CRP). Radiological reports, including chest X-rays, indicate any abnormalities such as opacities, infiltrates, etc.

Data analysis

Statistical analysis was performed using IBM SPSS Statistics version 25.0 (IBM, SPSS, Chicago, IL, USA). Demographic characteristics were presented as numbers. Odds ratios (OR) and Fisher's exact tests were used to assess the association between T2DM, dyslipidemia, and various parameters. Binary logistic regression was performed to determine the adjusted OR for these variables. The significance level of P < 0.05 was considered statistically significant.

Ethical considerations

The study protocol received approval from the Institutional Review Board (IRB) at Shaqra University, Saudia Arabia (IRB Number: 20-290C). Given the retrospective nature of the study involving existing medical records, the requirement for written informed consent was waived. Patient confidentiality was diligently maintained through coding and anonymization techniques.

## Results

Table 1 presents baseline characteristics of the study population, with a focus on the presence or absence of dyslipidemia. The data revealed several significant associations between dyslipidemia and various COVID-19 symptoms and outcomes. Fever is notably linked to dyslipidemia (P = 0.048), indicating that patients with dyslipidemia are more likely to experience it during their illness. Furthermore, tachypnea is strongly associated with dyslipidemia (P = 0.0001). Shortness of breath also exhibits a significant association with dyslipidemia (P = 0.011). Additionally, T2DM and ICU admission are strongly linked to dyslipidemia (P = 0.0001 and P = 0.001), respectively.

**Table 1:** Baseline characteristics of the study population (n = 239) *Significant associations are indicated by a P-value of <0.05. P-value reported using Fisher’s exact test. T2DM: type 2 diabetes mellitus, ICU: intensive care unit.

Characteristics	Dyslipidemia		P-value
	No, n (%)	Yes, n (%)	
Cough			
Yes	43 (22.52%)	148 (77.48%)	0.162
No	6 (12.50%)	42 (87.50%)	
Fever			
Yes	44 (23.15%)	146 (76.85%)	0.048*
No	5 (10.20%)	44 (89.80%)	
Tachypnea			
Yes	44 (32.35%)	92 (67.64%)	0.0001*
No	5 (4.85%)	98 (95.15%)	
Chest pain			
Yes	4 (22.22%)	14 (77.78%)	0.769
No	45 (20.36%)	176 (79.64%)	
Shortness of breath			
Yes	43 (24.57%)	132 (75.42%)	0.011*
No	6 (9.37%)	58 (90.63%)	
T2DM			
Yes	47 (33.34%)	94 (66.66%)	0.0001*
No	2 (2.04%)	96 (97.96%)	
ICU admission			
Yes	28 (31.81%)	60 (68.18%)	0.001*
No	21 (13.91%)	130 (86.09%)	

Table 2 provides a binary logistic analysis of baseline characteristics between patients with and without dyslipidemia who have COVID-19 infection. The results revealed several significant associations. T2DM and dyslipidemia are almost coexistent with an extremely high value of adjusted OR (aOR = 120.82, P = 0.0001). Tachypnea and SoB exhibited a very significant association with dyslipidemia (aOR = 232.1 (15.207-3,542.457), P = 0.000; and 10.729 (1.251-92.016), P = 0.003, respectively). Similarly, ICU admission is also significantly associated with dyslipidemia, both independently (OR = 1.810 (1.314-2.493), as well as in presence of T2DM (aOR = 8.136 (1.280-51.713), P = 0.026). Interesting to note is that T2DM independently was not associated with ICU setting (adjusted OR = 1.159, 95% CI: 0.539-2.496, P = 0.705), indicating more importance of former than latter for the same. In contrast, other symptoms, including cough, fever, and chest pain, do not show significant associations with dyslipidemia in binary logistic regression analysis.

**Table 2:** Binary logistic analysis of baseline characteristics between dyslipidemia and non-dyslipidemia patients with COVID-19 infection (n = 239) *Significant associations are indicated by a P-value of <0.05. Fisher’s exact test was used to calculate P-values. aOR: adjusted OR, T2DM: type 2 diabetes mellitus, ICU: intensive care unit.

Characteristics	Dyslipidemia		OR (95% CI)	aOR (95% CI)	P-value
	No, n (%)	Yes, n (%)			
Cough					
Yes	43 (22.52%)	148 (77.48%)	0.991 (0.904-1.088	-	-
No	6 (12.50%)	42 (87.50%)			
Fever					
Yes	44 (23.15%)	146 (76.85%)	1.169 (1.034-1.321)	0.320 (0.072-1.426)	0.135
No	5 (10.20%)	44 (89.80%)			
Tachypnea					
Yes	44 (32.35%)	92 (67.65%)	1.854 (1.558-2.208)	232.1 (15.207-3,542.457)	0.000*
No	5 (4.85%)	98 (95.15%)			
Chest pain					
Yes	4 (22.22%)	14 (77.78%)	0.554 (0.250-1.227)	1.781 (0.386-8.211)	0.459
No	45 (20.36%)	176 (79.64%)			
Shortness of breath					
Yes	43 (24.57%)	132 (75.43%)	1.263 (1.097-1.454)	10.729 (1.251-92.016)	0.030
No	6 (9.37%)	58 (90.63%)			
T2DM					
Yes	47 (33.34%)	94 (66.66%)	1.939 (1.661-2.264)	120.82 (9.342-1,562.482)	0.001*
No	2 (2.04%)	96 (97.96%)			
ICU admission					
Yes	28 (32.55%)	60 (67.45%)	1.810 (1.314-2.493)	8.136 (1.280-51.713)	0.026*
No	21 (13.90%)	130 (86.10%)			

Table 3 presents the associations between lipid profile (LDL, HDL, triglycerides, and total cholesterol) and the likelihood of ICU admission in COVID-19 patients. The results show significant association both independently, and in presence of T2DM as covariate with HDL cholesterol, triglycerides, and total cholesterol (P < 0.05 for all). In contrast, low-density lipoprotein (LDL) levels did not show statistically significant associations with ICU admission. These findings suggest that specific lipid profiles, particularly low HDL and elevated triglycerides, may be relevant factors in predicting the need for ICU care in COVID-19 patients.

**Table 3:** Associations between lipid profiles and ICU admission (n = 172) *Significant associations are indicated by a P-value of <0.05. Lipid profiles are categorized according to clinical thresholds. ICU: intensive care unit, LDL: low-density lipoprotein, HDL: high-density lipoprotein, TGI: triglyceride-glucose index.

Characteristics	ICU admission		OR (95% CI)	P-value	Adjusted OR (95% CI)	P-value
	No, n (%)	Yes, n (%)				
LDL						
Optimal <3.36	66 (57.39%)	49 (42.60%)	1		1	
High >4.11	10 (47.61%)	11 (52.39%)	1.48 (0.58-3.76)	0.47	0.546 (0.268-1.12)	0.096
Borderline 3.36-4.11	22 (61.11%)	14 (38.89%)	0.85 (0.39-1.84)	0.70	0.46 (0.28-1.12)	
HDL						
Low <1.03	86 (54.43%)	72 (45.57%)	1	0.02*	1	0.021*
Borderline 1.03-1.55	12 (85.71%)	2 (14.29%)	5.02 (1.08-23.18)		6.755 (1.327-34.27)	
TGI						
Optimal <1.69	23 (50.00%)	23 (50.00%)	1		1	
High >2.25	20 (36.36%)	35 (63.64%)	1.75 (0.78-3.88)	0.22	1.851 (1.228-2.791)	0.003*
Borderline 1.69-2.25	27 (62.79%)	16 (37.21%)	0.59 (0.25-1.38)	0.28	1.51 (1.22-2.91)	
Total cholesterol						
Optimal <5.2	88 (59.45%)	60 (40.54%)	1		1	0.05*
High >6.2	1 (20.00%)	4 (80.00%)	5.86 (0.63-53.78)	0.16	3.110 (0.966-9.710)	
Borderline 5.3-6.2	9 (47.36%)	10 (52,64%)	1.62 (0.62-4.24)	0.32	3.11 (0.96-0.71)	

Table 4 focuses on laboratory and inflammatory marker results between patients with and without dyslipidemia. There is a very strong association of fibrinogen with dyslipidemia in the presence of T2DM, in the context of ICU setting (1,235 (10.021-152,261.746), P = 0.004). Chest X-ray (CXR) findings also showed significant association with dyslipidemia (P = 0.039).

**Table 4:** Laboratory and inflammatory marker results between dyslipidemia and non-dyslipidemia *Significant associations are indicated by a P-value of <0.05. Fisher's exact test was used for analysis. CRP: C-reactive protein; CXR: chest X-ray.

Characteristics	Dyslipidemia		OR (95% CI)	Fisher’s exact 2-sided P-value	Adjusted OR (95% CI)	P-value
	No, n (%)	Yes, n (%)				
Fibrinogen						
Normal	5 (20.00%)	20 (80.00%)	0.634 (0.255-1.577)	0.464	1,235.0 (10.021-152,261.746)	0.004*
Abnormal	36 (30.00%)	84 (70.00%)	1		1	
D-dimer						
Normal	9 (19.56%)	37 (80.44%)	0.783 (0.413-1.483)	.545	0.665 (0.202-2.185)	0.501
Positive	32 (25.19%)	95 (74.81%)	1		1	
CRP						
Normal	21 (22.58%)	72 (77.42%)	0.988 (0.732-1.333)	1.000	0.191 (0.060-.605)	0.005
Abnormal	25 (21.18%)	88 (74.57%)	1		1	
CXR						
Clear	6 (10.71%)	50 (89.29%)	1.191 (1.041-1.363)	0.039*	0.044 (0.002-0.856)	0.039
Infiltrate (mostly bilateral)	43 (23.49%)	140 (76.51%)	1		1	

## Discussion

The results of this study present a comprehensive analysis of the associations between dyslipidemia, T2DM, and various clinical outcomes in patients with COVID-19 in Riyadh, Kingdom of Saudi Arabia (KSA). These findings shed light on the intricate relationship between these comorbid conditions and the severity of COVID-19. Several key observations emerged from the analysis.

First and foremost, the data showed a strong association between dyslipidemia and T2DM, underscoring their interdependence (aOR = 120.82, P = 0.0001). This finding aligns with previous research that has established a close relationship between T2DM and dyslipidemia due to shared risk factors and pathophysiological mechanisms, including chronic inflammation and endothelial dysfunction [15,16]. Moreover, the prevalence of T2DM in Saudi Arabia is among the highest globally, emphasizing the significance of understanding its impact on COVID-19 prognosis in this specific population [17,18].

The presence of tachypnea and SoB was significantly associated with dyslipidemia. This observation suggests that patients with dyslipidemia are more likely to exhibit this symptom, which can be indicative of respiratory distress and is a marker of severe COVID-19 cases [19,20]. The potential link between dyslipidemia and respiratory distress warrants further investigation, as it may have implications for patient management strategies.

Intensive care unit (ICU) admission was also notably associated with dyslipidemia independently, as well as in the presence of T2DM (aOR = 8.136 (1.280-51.713), P = 0.026). The association between lipid profile and intensive care unit (ICU) admission in COVID-19 patients reveals noteworthy insights into the potential role of dyslipidemia in shaping disease severity and clinical outcomes. Notably, reduced levels of high-density lipoprotein (HDL), elevated triglyceride, and total cholesterol were significantly associated both independently and in the presence of T2DM as a confounding factor. This finding underscores the heightened risk that patients with dyslipidemia face in terms of requiring critical care, which is an important factor in healthcare resource allocation and patient outcomes. The strong association between dyslipidemia and the need for ICU admission may be related to the impact of dyslipidemia on the immune system and inflammatory responses [16-20].

In contrast, other common COVID-19 symptoms, such as cough, fever, and chest pain, did not exhibit significant associations with dyslipidemia. This emphasizes the specificity of the observed connections between dyslipidemia and certain clinical outcomes. These results are in line with the multifaceted nature of COVID-19, where various factors, including comorbid conditions, contribute to the clinical picture [21].

Additionally, fibrinogen and chest X-ray (CXR) findings displayed a significant association with ICU admission (P = 0.039), indicating that patients with dyslipidemia are more likely to exhibit fibrosis and abnormalities on CXR. These abnormalities, particularly infiltrates, are often associated with more severe COVID-19 cases. When adjusting for other factors, this association became even more significant. The odds ratio of 2.993 (95% CI: 0.490-0.856) for CXR abnormalities, along with the pronounced aOR of 1,235.23 (95% CI: 10.021-152,261.746) for fibrinogen values, underscores a higher probability of ICU admission and can contribute to the overall severity of the disease. This suggests that patients with dyslipidemia are more likely to have abnormal fibrinogen levels, indicative of an activated inflammatory response. The connection between dyslipidemia and elevated fibrinogen levels aligns with existing research, which has identified a link between dyslipidemia and systemic inflammation [21,22].

The findings of this study hold significant implications for healthcare management in Riyadh, KSA, and potentially in other regions facing similar challenges. The strong association between dyslipidemia, T2DM, and the severity of COVID-19 emphasizes the need for comprehensive risk assessment and tailored care plans for patients with these comorbid conditions, especially in the context of ICU admissions. Early identification and management of dyslipidemia in patients with T2DM could potentially mitigate the risk of severe COVID-19 and the need for ICU admission. Furthermore, these results can inform public health policies and resource allocation, aiding healthcare providers and policymakers in optimizing their response to the COVID-19 pandemic.

The study's implications for clinical practice and public health highlight the importance of proactive risk assessment and tailored care for patients with comorbid conditions, particularly dyslipidemia with or without T2DM. Healthcare providers should focus on optimizing the management of these conditions, given their significant impact on COVID-19 severity. Early intervention, close monitoring of respiratory distress symptoms, and allocation of resources, such as intensive care unit (ICU) admission, based on comorbid conditions are critical aspects of clinical practice. In the realm of public health, messaging and preventive programs can emphasize the importance of managing comorbidities, while resource allocation strategies and continuous health education can enhance pandemic preparedness and improve overall outcomes.

Strength

This study has several strengths, including a clearly defined population from a tertiary hospital during the early phase of the COVID-19 pandemic, use of detailed clinical and biochemical data, and application of robust statistical methods such as multivariable logistic regression. These contribute to the reliability and internal validity of our findings.

Limitation

Despite its strengths, the study has a few limitations that should be considered. Being retrospective in nature, it is subject to potential selection bias, as only hospitalized patients were included, potentially excluding milder or asymptomatic cases. Additionally, we could not account for unmeasured confounders such as obesity, smoking status, physical activity, and socioeconomic variables, which may have influenced both disease severity and ICU admission rates. Despite statistical adjustments, the possibility of residual confounding cannot be entirely ruled out. Finally, as a single-center study, the findings may not be generalizable to other populations or healthcare settings.

## Conclusions

This study concluded that dyslipidemia independently, as well as in conjunction with T2DM, is significantly associated with increased risks of respiratory distress and intensive care unit (ICU) admission in COVID-19 patients in Riyadh, KSA. These findings underscore the need for tailored care and resource allocation strategies for comorbid patients and highlight the multifaceted nature of COVID-19. It has critical implications for clinical practice and public health, enhancing pandemic preparedness and patient outcomes in Riyadh and similar regions.
